# 

**Published:** 1829

**Authors:** 


### CONTENTS OF NO. II, VOL. III.

### ESSAYS AND CASES.

Art. I.—An Account of the Autumnal Fever of Georgia, from observations made during four years, in the counties of Jones and Baldwin. By James C. Finley, M. D. now of Cincinnati. - - - - - - - - 165

II.—An account of a fatal case of Acute Negro Consumption, with the appearances on dissection. By Dr. John Bennett, of Newport, Kentucky. - - - - - 193

III.—Memoranda of a supposed case of Ruptured Uterus consequent upon a partial adhesion of the Vaginal parieties. By Richard F. Barrett, M. D. of Greensburg, Kentucky. 196

IV.—A case of Epilepsy suspended by a Burn on the sole of the foot. Communicated by Dr. H. E. Green, of Greenupsburgh, Kentucky. - - - - - - 199

V.—Notes on a case of Spina Bifida. By the Editor. 200

VI.—An account of a singular case of Fœtal Monstrosity. By Dr. John Cook Bennett, of McConnellsville, Ohio. 213

VII.—Medical Jurisprudence. Sequel of the case of John Birdsell, with remarks on Feigned Insanity. By the Editor. 215

### REVIEWS AND BIBLIOGRAPHICAL NOTICES.

Art. VIII. Lænnec on Diseases of the Chest. Philadelphia, 1823.

Collin on the Stethoscope, Boston, 1829. , , 222

### MISCELLANEOUS INTELLIGENCE.

### I. Analectic.

1. On the condition of the Blood and Blood-vessels in Inflammation. - - - , , , 261
2. On the Coagulation of the Blood. , , 264
3. On the Therapeutic properties of Morphine. , 264
4. On Strangury from Cantharides, and its relief. , 267
5. On the tests by which Morphine may be made known in cases of Poisoning. - - - - 268
6. On the Influence of Mercury on the functions of the Uterus. 269
7. Cataract and Glaucoma. - - - 270

8. On small doses of oil of Turpentine for the cure of sciatica and other neuralgiæ. - - - - 271
9. On the form of the face and forehead; with a theory of their formation in different ages and nations. , , 272
10. Pathology of Muscæ Volitantes. , , 274
11. Tartar on the Teeth. - , , 275
12. New mode of administering Quinine. , - 276
13. On the supposed Existence of Active Molecules in Mineral substances. - - - - 276
14. On the Physiological Effects of Oxygen Gas, upon the Animal System. - - - - - - 279
15. Silver found in the viscera of a person who had used the Nitrate of Silver. . , , , 280
16. Rhubarb plant. , , . - 280
17. On the Treatment of Intermittent Fevers by the Sulphate of Quinine, applied to a denuded surface. , , 280
18. Ergot of Rye in Chronic Uterine Discharges. , 281
19. Talicotian Operation performed with success. , 282
20. Fungous Tumours from the Gum and Alveoli. , , 282
21. Small Pox. , , , , 283
22. Mercury detected in Swaim's Panacea, by Chemical analysis. 285
23. Some account of the Siamese boys, lately brought to Boston. 286
24. Extract from another account of the same. , , 288
25. A case of twins united by the Umbilicus. By Joshua Martin, M. D. of Xenia, Ohio. , , , 290
26. General Phlebitis. , , , , 291
27. Varieties of Hydrocele—Modifications of the usual operations. , , , , , 292
28. On the Reduction of Herniæ. By M. Dupuytren. 293
29. Supposed Artificial Diamonds. , , , 295

### II. Analytical and Original.

30. Wounds of the Heart. Prof. Coxe. , , 295
31. Respiration of Children in Reversed Presentations. By Professor Bigelow. , , , , , 299
31. Aneurism. Tying the artery on the distal side. Mr. Wardrop. 301
32. Oil of Turpentine in incarcerated Herniæ. Prof. Sewall. 303
33. Dengue. By Dr. Daniell. , , , , 304
34. Modus operandi of poisons. , , , , 305
35. Morbid appearances after death, from Colica Pictonum. By E. J. Coxe, M. D. , , , , 312
36. Silliman's Journal. , , , , , 313
37. To our Readers. , , , , , , 314

